# Supplementary material for: An H3K9 methylation-dependent protein interaction regulates the non-enzymatic functions of a putative histone demethylase
Source: eLife. 2020 Mar 20;9:e53155. doi: 10.7554/eLife.53155 (PMC7192584; doi:10.7554/eLife.53155)
Supplement: Supplementary file 1. [file elife-53155-supp1.docx]

**Supplemental File 1. Strains used in this study**

| **Strains** | **Strains description** | **Strain sources** | **Related to** |
| --- | --- | --- | --- |
| KR18 | *h^-^ leu1-32 ade6 M210 ura4Δ::10XTetO-ade6^+^ clr4^+^ trp1:natMX6-clr4p-TetR-clr4ΔCD* | Moazed lab | Figure 1 B-D, 3C, S3H |
| KR33 | *h^-^ leu1-32 ade6? ura4Δ::10XTetO- ade6^+^ clr4^+^ trp1:natMX6-clr4p-TetR-clr4ΔCD epe1Δ::kanMX6* | Moazed lab | Figure 1 B-D, 3C, S3H |
| KR283 | *h^-^ leu1-32 ade6 M210 ura4Δ::10XTetO- ade6^+^ clr4^+^ trp1:natMX6-clr4p-TetR-clr4ΔCD 3XFLAG-epe1-hphMX6 (reinsertion)* | This study | Figure 1B-D |
| KR303 | *h^-^ leu1-32 ade6 M210 ura4Δ::10XTetO - ade6^+^ clr4^+^ trp1:natMX6-clr4p-TetR-clr4ΔCD 3XFLAG-epe1 H297A -hphMX6* | This study | Figure 1B-D, 3C |
| KR844 | *h^-^ leu1-32 ade6 M210 ura4Δ::10XTetO - ade6^+^ clr4+ trp1:natMX6-clr4p-TetR-clr4ΔCD 3XFLAG-epe1 Y307A -hphMX6* | This study | Figure 1B-D, 3C |
| KR846 | *h^-^ leu1-32 ade6 M210 ura4Δ::10XTetO - ade6^+^ clr4^+^ trp1:natMX6-clr4p-TetR-clr4ΔCD 3XFLAG-epe1 Y370A-hphMX6* | This study | Figure 1B-D |
| KR24 | *h^-^ leu1-32 ade6 M210 ura4Δ::10XTetO- ade6^+^ clr4Δ::kanMX6* | Moazed lab | Figure 1C-D, 5E-F, S5B-C,F-G |
| KR817 | *h^90^ ade6-M216 leu1-32 ura4-D18 mCherry-v5-Swi6 mNeonGreen-Epe1 WT-hphMX6* | This study | Figure 1E |
| KR821 | *h^90^ ade6-M216 leu1-32 ura4-D18 mCherry-v5-Swi6 mNeonGreen-Epe1 Y307A-hphMX6* | This study | Figure 1 F |
| KR342 | *h^+^ leu1-32 otr1R(SphI)::ura4^+^ ura4-DS/E ade6 M210* | Grewal lab | Figure S1B, 2A-B,  S2B, 4A-B, S4A, 5A |
| KR471 | *h^-^ leu1-32 epe1-3XFLAG-hphMX6* | This study | Figure S1B, 2A-B,  S2B, 4A-B, S4A, 5B, 6B |
| KR128 | *h^-^ leu1-32 epe1-H297A-3XFLAG-hphMX6* | This study | Figure S1B, 2A-B, S2B, 5B, 6B |
| KR769 | *h^+^ leu1-32 ura4D18 imr1R(Ncol)::ura4^+^ oriL ade6 M216 epe1 Y307A-3XFLAG-hphMX6* | This study | Figure S1B, 2A-B,  S2B |
| KR705 | *h^+^ otr1R(SphI)::ura4^+^ ura4-DS/E leu1-32 ade6 M210 epe1 Y370A -3XFLAG-hphMX6* | This study | Figure S1B, 2A-B, S2B |
| KR132 | *h^-^ leu1-32 epe1-3XFLAG-hphMX6 swi6Δ::kanMX6* | This study | Figure 2B, S2B, 5B, 6B |
| KR279 | *h+ leu1-32 ura4-D18 imr1R(NCol)::ura4+ oriI ade6-216 3XFLAG-epe1-hphMX6* | This study | Figure S2A |
| KR285 | *h+ leu1-32 ura4-D18 imr1R(NCol)::ura4+ oriI ade6-216 3XFLAG-epe1 H297A-hphMX6* | This study | Figure S2A |
| KR947 | *h- leu1-32 3XFLAG-NLS-epe1 1-434 WT hphMX6* | This study | Figure 3B, S3F |
| KR1197 | *h^-^ leu1-32 ade6? ura4Δ::10XTetO- ade6^+^ clr4^+^ trp1:natMX6-clr4p-TetR-clr4ΔCD 3XFLAG-NLS-epe1 434-948-hphMX6 (JmjCΔ) #1* | This study | Figure 3C-D |
| KR1198 | *h^-^ leu1-32 ade6? ura4Δ::10XTetO- ade6^+^ clr4^+^ trp1:natMX6-clr4p-TetR-clr4ΔCD 3XFLAG-NLS-epe1 434-948-hphMX6 (JmjCΔ) #2* | This study | Figure 3C-D |
| KR1199 | *h^-^ leu1-32 ade6? ura4Δ::10XTetO- ade6^+^ clr4^+^ trp1:natMX6-clr4p-TetR-clr4ΔCD 3XFLAG-NLS-epe1 434-948-hphMX6 (JmjCΔ) #3* | This study | Figure 3C-D |
| KR862 | *h^-^ leu1-32 ade6 M210 ura4Δ::10XTetO- ade6^+^ clr4^+^ trp1:hphMX6-swi6p-TetR-Swi6 CSD epe1+* | This study | Figure 3E-G S3H-I |
| KR865 | *h^-^ leu1-32 ade6 M210 ura4Δ::10XTetO- ade6^+^ clr4^+^ trp1:hphMX6-swi6p-TetR-Swi6 CSD clr4Δ::kanMX6* | This study | Figure 3F-G |
| KR866 | *h^-^ leu1-32 ade6 M210 ura4Δ::10XTetO- ade6^+^ clr4^+^ trp1:hphMX6-swi6p-TetR-Swi6 CSD epe1Δ::kanMX6 #1* | This study | Figure 3E-G S3H-I |
| KR867 | *h^-^ leu1-32 ade6 M210 ura4Δ::10XTetO- ade6^+^ clr4^+^ trp1:hphMX6-swi6p-TetR-Swi6 CSD epe1Δ::kanMX6 #2* | This study | Figure S3H |
| KR868 | *h^-^ leu1-32 ade6 M210 ura4Δ::10XTetO- ade6^+^ clr4^+^ trp1:hphMX6-swi6p-TetR-Swi6 CSD epe1Δ::kanMX6 #3* | This study | Figure S3H |
| KR1365 | *h^-^ leu1-32 ade6 M210 ura4Δ::10XTetO- ade6^+^ clr4^+^ trp1:bsdMX6-swi6p-TetR-Swi6 CSD 3XFLAG-NLS-epe1 434-948-hphMX6 (JmjCΔ) #1* | This study | Figure 3E-G |
| KR1366 | *h^-^ leu1-32 ade6 M210 ura4Δ::10XTetO- ade6^+^ clr4^+^ trp1:natMX6-swi6p-TetR-Swi6 CSD 3XFLAG-NLS-epe1 434-948-hphMX6 (JmjCΔ) #2* | This study | Figure 3E-G |
| KR1367 | *h^-^ leu1-32 ade6 M210 ura4Δ::10XTetO- ade6^+^ clr4^+^ trp1:natMX6-swi6p-TetR-Swi6 CSD 3XFLAG-NLS-epe1 434-948-hphMX6 (JmjCΔ) #3* | This study | Figure 3E |
| KR1368 | *h^-^ leu1-32 ade6 M210 ura4Δ::10XTetO- ade6^+^ clr4^+^ trp1:natMX6-swi6p-TetR-Swi6 CSD 3XFLAG-NLS-epe1 434-948-hphMX6 (JmjCΔ) #4* | This study | Figure 3E |
| KR1227 | *h^-^ leu1-32 3XFLAG-NLS-epe1 434-948-hphMX6 (JmjCΔ)* | This study | Figure S3D |
| KR1357 | *h- leu1-32 3XFLAG-NLS-epe1 1-434 WT hphMX6 swi6Δ* | This study | Figure S3G |
| KR1210 | *h- leu1-32 3XFLAG-NLS-epe1 1-434 H297A hphMX6* | This study | Figure S3F |
| KR1346 | *h- leu1-32 3XFLAG-NLS-epe1 1-434 H297A hphMX6 swi6Δ* | This study | Figure S3G |
| KR869 | *h^-^ leu1-32 ade6 M210 ura4Δ::10XTetO- ade6^+^ clr4^+^ trp1:hphMX6-swi6p-TetR-Swi6 CSD clr4Δ::natMX6 epe1Δ::kanMX6* | This study | Figure S3I |
| KR228 | *h^+^ otr1R(SphI)::ade6^+^ ura4Δ18 leu1-32 ade6 M210 H3.1/H3.2/H3.3 K9R epe13X-FLAG-hphMX6* | Moazed lab | Figure 4A |
| KR130 | *h^-^ leu-32 epe1 3X-FLAG-hphMX6 clr4Δ::kanMX6* | This study | Figure 2B, S2B, 4A, S5E, 6B |
| KR724 | *h^-^ leu-32 epe1 3X-FLAG hphMX6 clr3Δ::kanMX6* | This study | Figure 4B |
| KR726 | *h^-^ leu-32 epe1 H297A 3X-FLAG hphMX6 clr3Δ::kanMX6* | This study | Figure 4B |
| KR728 | *h^-^ leu-32 epe1 3X-FLAG-hphMX6 sir2Δ::kanMX6* | This study | Figure 4B |
| KR730 | *h^-^ leu-32 epe1 H297A 3X-FLAG-hphMX6 sir2Δ::kanMX6* | This study | Figure 4B |
| KR732 | *h^-^ leu1-32 ade6? ura4D18 mst2Δ::kanMX6 epe1 3X-FLAG-hphMX6* | This study | Figure S4A |
| KR734 | *h^-^ leu1-32 ade6? ura4D18 mst2Δ::kanMX6 epe1 H297A 3X-FLAG-hphMX6* | This study | Figure S4A |
| KR945 | *h- leu1-32 NLS-3XFLAG-epe1-ΔC-hphMX6* | This study | Figure S5D-E |
| KR1361 | *h- leu1-32 NLS-3XFLAG-epe1-ΔC-hphMX6 clr4Δ::kanMX6* | This study | Figure S5E |
| KR906 | *h^-^ leu1-32 epe1 3X-FLAG hphMX6 clr3 3X-V5-natMX6* | This study | Figure 6A |
| KR908 | *h^-^ leu1-32 epe1 H297A-3XFLAG-hphMX6 clr3 3X-V5-natMX6* | This study | Figure 6A |
| KR675 | *h^-^ ade6 M210 ura4Δ::10XGal4-10XTetO- ade6^+^ natMX6-TetR-clr4-I epe1^+^ leu1^+^:nmt1-gal4-sir2* | This study | Figure 6G-I |
| KR677 | *h^-^ ade6 M210 ura4Δ::10XGal-10XTetO-ade6^+^ natMX6-TetR-clr4-I epe1^+^ leu1^+^:nmt1-gal4-clr3* | This study | Figure 6G-I. S5A-C,F-G |
| KR606 | *h^-^ leu1-32 ade6 M210 ura4Δ::10XGal4-10XTetO-ade6^+^ trp1:natMX6-TetR-clr4-I* | This study | Figure S6A, |
| KR643 | *h^-^ leu1-32 ade6 M210 10XTetO- ade6^+^ natMX6-TetR-Clr4-I epe1Δ::kanMX6* | This study | Figure 6G |
| KR1075 | *h^-^ ade6 M210 ura4Δ::10XGal4-10XTetO-ade6^+^ leu1^+^:nmt1-gal4-clr3* | This study | Figure S6A-C |
| KR1071 | *h^-^ ade6 M210 ura4Δ::10XGal-10XTetO- ade6^+^  natMX6-TetR-clr4-I epe1^+^ leu1^+^:nmt1-clr3 (-gal4)* | This study | Figure S6A-C |
| KR318 | *h+ leu1-32 ura4-D18 imr1R(NCol)::ura4+ oriI ade6-216* | Grewal lab | Figure 6C-E |
| KR267 | *h+ leu1-32 ura4-D18 imr1R(NCol)::ura4+ oriI ade6-216 epe1-3XFLAG-hphMX6* | This study | Figure 6C-E |
| KR1397 | *h+ leu1-32 ura4-D18 imr1R(NCol)::ura4+ oriI ade6-216 epe1-3XFLAG-hphMX6 clr3Δ::kanMX6* | This study | Figure 6C-E |
| KR1103 | *h^-^ ade6 M210 ura4Δ::10XGal4-10XTetO- ade6^+^ natMX6-TetR-clr4-I epe1^+^ leu1^+^:nmt1-gal4-clr3 dcr1Δ:: kanMX6* | This study | Figure S6D |
