# Supplementary material for: An H3K9 methylation-dependent protein interaction regulates the non-enzymatic functions of a putative histone demethylase
Source: eLife. 2020 Mar 20;9:e53155. doi: 10.7554/eLife.53155 (PMC7192584; doi:10.7554/eLife.53155)
Supplement: Supplementary file 2. [file elife-53155-supp2.docx]

**Supplementary File 2.** Plasmids used in this study

| **Plasmid name** | **Backbone** | **Features** | **Reference** | **Figures** |
| --- | --- | --- | --- | --- |
| pKR525 | pFA6a natMX6 TetR-Clr4-I | 2X FLAG inserted between TetR and Clr4-I | Ragunathan et al., 2015 | Figures |
| pKR389 | pFA6a-hphMX6- TetR-2XFLAG Swi6-CSD | 2X FLAG tag inserted between TetR and the Swi6 CSD domain | This study | Figure 3E-G, Figure 3-figure supplement 3H-I |
| pKR174 | pGEX-6p-1 3XFLAG-Swi6 | 3XFLAG inserted downstream of prescission protease site | This study | Figure 2, 3, 4, 5 |
| pKR589 | pGEX-6p-1 3XFLAG-Chp2 | 3XFLAG inserted downstream of prescission protease site | This study | Figure 2C |
| pKR593 | pFASTBAC 6XHis-MBP-TEV Epe1 | Epe1 full length | This study | Figure 2,3,4,5 |
| pKR594 | pFASTBAC 6XHis-MBP-TEV Epe1 H297A | Epe1 full length | This study | Figure 2XX, Figure 5-figure supplement 5XX |
| pKR357 | pFASTBAC 6XHis-MBP-TEV Epe1-ΔC | Epe1 truncation allele | This study | Figure 5 |
| pKR499 | pET 6XHis- MBP-TEV-Epe1 434-948aa | Epe1 C-terminal fragment | This study | Figure 3A-B, Figure 5B |
| pKR500 | pET 6XHis- MBP-TEV-Epe1 434-600aa | Epe1 C-terminal fragment | This study | Figure 3- figure supplement 3XX |
| pKR498 | pGEX-6p-1 3XFLAG-Swi6 L315E | Swi6 chromoshadow domain mutant | This study | Figure 2C |
| pKR595 | pGEX-6p-1 3XFLAG-Swi6 Loop-X mutant | Swi6 oligomerization mutant | This study | Figure 4- figure supplement 4I |
| pKR413 | pGEX-6p-1 3XFLAG-Swi6 W104A | Swi6 chromodomain mutant | This study | Figure 4E |
| pKR237 | pET28a-6XHis-SUMO-Swi6 | Purify Swi6 without any tag | This study | Figure 3B |
